# Detection level and pattern of positive lesions using PSMA PET/CT for staging prior to radiation therapy

**DOI:** 10.1186/s13014-017-0902-0

**Published:** 2017-11-10

**Authors:** Nina-Sophie Schmidt-Hegemann, Wolfgang Peter Fendler, Alexander Buchner, Christian Stief, Paul Rogowski, Maximilian Niyazi, Chukwuka Eze, Minglun Li, Peter Bartenstein, Claus Belka, Ute Ganswindt

**Affiliations:** 1 Department of Radiation Oncology, University Hospital, LMU Munich, Munich, Germany; 2 Department of Nuclear Medicine, University Hospital, LMU Munich, Munich, Germany; 3 Department of Urology, University Hospital, LMU Munich, Munich, Germany; 4 Department of Therapeutic Radiology and Oncology, Innsbruck Medical University, Anichstr. 35, A-6020 Innsbruck, Austria

**Keywords:** ^68^Ga-PSMA PET/CT, Prostate cancer, Radiotherapy, Radical prostatectomy

## Abstract

**Background:**

To determine the potential role of ^68^Ga-PSMA positron emission tomography/computed tomography (PET/CT) in radiotherapy (RT) planning for prostate cancer (PCa).

**Methods:**

One hundred twenty-nine patients (pts) with ^68^Ga-PSMA PET/CT were retrospectively analysed. Potentially influencing factors (androgen deprivation therapy, amount of ^68^Ga-PSMA-HBED-CC, PSA doubling time ≤/> 10 months, PSA before PET/CT, T−/N-category and Gleason score) were evaluated by logistic regression analysis. The detection rate of PSMA PET/CT was compared to contrast enhanced CT and its impact on RT management analysed.

**Results:**

One hundred twenty-nine patients (pts) (20 at initial diagnosis, 49 with PSA relapse and 60 with PSA persistence after radical prostatectomy) received PSMA PET/CT prior to RT. The majority of pts. (71.3%) had PET-positive findings (55.1% of pts. with PSA recurrence, 75% of pts. with PSA persistence and 100% of newly diagnosed pts). Median PSA before PET/CT in pts. with pathological findings (*n* = 92) was 1.90 ng/ml and without (*n* = 37) 0.30 ng/ml. PSA level at time of PET/CT was the only factor associated with PET-positivity. In pts. with a PSA ≤ 0.2 ng/ml, the detection rate of any lesion was 33.3%, with a PSA of 0.21–0.5 ng/ml 41.2% and with a PSA of 0.51–1.0 ng/ml 69.2%, respectively. Regarding the anatomic distribution of lesions, 42.2% and 14.7% of pts. with relapse or persistence had pelvic lymph node and distant metastases. In pts. at initial diagnosis the detection rate of pelvic lymph nodes and distant metastases was 20% and 10%. ^68^Ga-PSMA PET/CT had a high detection rate of PCa recurrence outside the prostatic fossa in pts. being considered for salvage RT (22.4% PET-positive pelvic lymph nodes and 4.1% distant metastases). Compared to CT, PSMA PET/CT had a significantly higher sensitivity in diagnosing rates of local recurrence/primary tumour (10.1% vs. 38%), lymph nodes (15.5% vs. 38.8%) and distant metastases (5.4% vs. 14.0%). This resulted in a modification of RT treatment in 56.6% of pts.

**Conclusions:**

The detection of PCa is strongly associated with PSA level at time of ^68^Ga-PSMA PET/CT. PSMA PET/CT differentiates between local, regional and distant metastatic disease with implications for disease management. PSMA PET/CT allows for tumour detection in post-prostatectomy pts. with PSA ≤ 0.5 ng/ml considered for salvage RT.

## Background

A precise detection and visualisation of disease extent is of essential importance for indication and target volume definition in radiotherapy of prostate cancer. Due to limitations in sensitivity and specificity, MRI and choline PET/CT are not considered precise enough especially at PSA levels ≤1 ng/ml [1, 2], although both imaging modalities are as well capable of modifying treatment approaches [3–7]. Recently, a ^68^Ga-labelled PSMA-targeted ligand with high affinity to prostate-specific membrane antigen (PSMA), a cell surface protein overexpressed in prostate cancer cells has been introduced for positron emission tomography (PET) imaging [8–11]. PET with ^68^Ga-PSMA demonstrated a superior tumour-to-background signal intensity and substantially higher detection rates than have been previously reported for other imaging modalities in patients with newly diagnosed [12–15] or recurrent [3, 16–20] prostate cancer. From surgical series, it is known that ^68^Ga-PSMA positron emission tomography/computed tomography (PET/CT) allows for a highly correct identification of lymph node metastases with a sensitivity and specificity ranging from 66% to 84% and 82% to 99% [14, 15], respectively. However, there is a substantial percentage of prostate cancer patients with a negative ^68^Ga-PSMA PET/CT. Bearing this in mind, we tried to determine retrospectively which factors correlate with a positive ^68^Ga-PSMA PET/CT in patients with primary, persistent or recurrent prostate cancer. Furthermore, we compared the detection rate of ^68^Ga-PSMA PET/CT to contrast enhanced CT and analysed its impact on the decision-making process within our department of radiation oncology. Several clinical scenarios may be distinguished: A PET-positive local recurrence within the prostatic fossa might justify a simultaneous integrated boost and an additional antiandrogen therapy. Likewise, PET-positive pelvic lymph node metastases might be treated with an enlargement of the clinical target volume, a focally higher dose and antiandrogen therapy. In addition, the evidence of distant metastases might even cause a non-realisation of a planned radiotherapy treatment.

## Methods

### Study population

^68^Ga-PSMA PET/CT has been routinely offered to patients for prostate cancer staging before radiotherapy in our clinic (February 2014–August 2016). A total of 129 patients consecutively received ^68^Ga-PSMA PET/CT prior to radiotherapy in different clinical scenarios: 20 patients at initial diagnosis (mostly high-risk patients with suspected extra-prostatic manifestations), 60 with biochemical persistence after radical prostatectomy and 49 with biochemical relapse (Table 1). All patients gave written informed consent to undergo ^68^Ga-PSMA PET/CT. This retrospective analysis is in compliance with the principles of the Declaration of Helsinki and its subsequent amendments [21] and was approved by the Ethics Committee of the LMU Medical Faculty .

**Table 1:** Patients’ characteristics

Characteristic	All pts.	Initial diagnosis	PSA relapse	PSA persistence
Number	129	20	49	60
Age (years; median, range)	72 (47–86)	76 (53–86)	74 (50–83)	69 (47–83)
Gleason Score [pts]				
6	11 (8.5%)	3 (15%)	7 (14.3%)	1 (1.7%)
7a	27 (20.9%)	3 (15%)	19 (38.8%)	5 (8.3%)
7b	37 (28.7%)	6 (30%)	11 (22.4%)	20 (33.3%)
8	18 (14%)	3 (15%)	7 (14.3%)	8 (13.3%)
9	34 (26.4%)	5 (25%)	5 (10.2%)	24 (40.0%)
10	2 (1.6%)	–	–	2 (3.3%)
Risk group (D’Amico) [pts]				
low	5 (3.9%)	1 (5.0%)	3 (6.1%)	1 (1.7%)
intermediate	21 (16.3%)	2 (10.0%)	13 (26.5%)	6 (10.0%)
high	103 (79.8%)	17 (85%)	33 (67.3%)	53 (88.3%)
PSMA PET positive [pts]	92 (71.3%)	20 (100%)	27 (55.1%)	45 (75%)
PSA PRE-PSMA PET [ng/ml] (median/mean, range)	0.86/6.04 (0.13–150.00)	12.4/27.28 (0.14–150.0)	0.49/1.00 (0.15–6.24)	0.99/3.08 (0.13–39.2)
PSA doubling time				
≤ 10 months	81 (74.3%)	–	21 (42.9%)	60 (100%)
> 10 months	28 (25.7%)	–	28 (57.1%)	–
ADT at time of PET [pts]	14 (10.9%)	2 (10%)	4 (8.2%)	8 (13.3%)
Activity of 68Ga-PSMA-HBED-CC [MBq] (mean, range)	190.43 (87–293)	197.50 (100–293)	192.07 (94–293)	186.85 (87–286)

(*Pts* patients, *PSA* prostate specific antigen, *ADT* androgen deprivation therapy, *MBq* Mega-Becquerel)

### PSMA ligand and PET/CT imaging

PSMA-HBED-CC was radiolabelled with ^68^Ga^3+^ from a ^68^Ge/^68^Ga generator system (GalliaPharm^®^, Eckert & Ziegler AG, Berlin, Germany) using an automated synthesis module (GRP, Scintomics GmbH, Munich, Germany) and pre-packed cassettes (ABX GmbH, Radeberg, Germany) as described previously for a different PSMA ligand by Weineisen et al. [22]. ^68^Ga-PSMA PET/CT images extending from the base of the skull to the mid-thigh were acquired. PET/CT scan was obtained with intravenous injection of iodine-containing contrast agent (Ultravist 300, Schering, Berlin, Germany; or Imeron 300, Bracco, Konstanz; 2.5 mL/s; in portal venous phase) 60 min after almost simultaneous intravenous administration of 20 mg furosemide and ^68^Ga-PSMA (median 189 megabecquerel (MBq), range 87–293). Directly prior to the PET/CT scan, patients were asked to empty their bladder to minimise tracer accumulation.

### Image interpretation

PET/CT was interpreted by a consensus read of one nuclear medicine physician and one radiologist. Location of lesions was each determined by CT. PET-positive lesions were identified by ^68^Ga-PSMA uptake visually above background and not associated with the physiologic uptake. CT-positive nodes were defined by increased short axis diameter, loss of fatty hilum, or increased contrast enhancement. Bone metastases were detected by suspicious sclerotic lesions, visceral metastases by suspicious hypodense or hyperdense lesions in the respective organ. Based on PET/CT images and reports, stage according to PET or CT was documented separately by one nuclear medicine physician and one radiation oncologist [23].

### Statistical analysis

Demographic and tumour characteristics were analysed. The association between positive ^68^Ga-PSMA PET/CT findings and possibly interacting variables, like androgen deprivation therapy (ADT) at the time of PET/CT, amount of injected tracer, PSA-level, PSA doubling time, T−/N-category and Gleason score was assessed by univariate and multivariate logistic regression analysis. *P* values <0.05 were considered statistically significant. PSA before PET/CT was classified in eight steps (≤ 0.2 ng/ml – > 20 ng/ml). Gleason score was grouped in six different classes (GS 5/6, GS 7a, GS 7b, GS 8, GS 9 and GS 10), T-category in four (T1 – T4) and N-category in three classes (N0, N1, Nx). Injected tracer amount was evaluated as multiples of 100 MBq. Patients after radical prostatectomy were classified by PSA doubling time ≤ vs. > 10 months. PET/CT positive findings (primary tumour/local recurrence, lymph node and distant metastases) are presented separately according to the respective treatment indication prior to radiotherapy. The detection rate of residual tumour, recurrence or newly diagnosed disease comparing PET/CT to CT exclusively as well as change in radiotherapy management was analysed by Fisher’s test.

## Results

A total of 129 patients (20 at initial diagnosis, 60 with biochemical persistence and 49 with biochemical relapse after radical prostatectomy) consecutively received ^68^Ga-PSMA PET/CT prior to radiotherapy (Table 1). Most patients (71.3%) had ^68^Ga-PSMA PET/CT-positive findings: Patients with biochemical recurrence had the lowest detection rate (55.1%) followed by patients with biochemical persistence (75%). At initial diagnosis, all patients (100.0%) had ^68^Ga-PSMA PET/CT-positive findings. Overall, the predominant GS, T- and N-category were 7b, T3 and N0 – in newly diagnosed patients prior to definitive radiotherapy 7b, cT1/cT3 and cN0, in patients with biochemical relapse 7a, pT2 and pN0 and in patients with biochemical persistence 9, pT3 and pN1, respectively. Altogether, most patients had a high-risk prostate cancer (79.8%) according to D’Amico risk group classification [24]. PSA doubling time in post-prostatectomy patients differed between patients with biochemical recurrence having mainly a PSA doubling time > 10 months (57.1%) versus patients with biochemical persistence having a PSA doubling time ≤ 10 months (100%). ADT was in use in 14 patients at the time of ^68^Ga-PSMA PET/CT. Median PSA before ^68^Ga-PSMA PET/CT was 12.4 ng/ml in newly diagnosed patients, 0.99 ng/ml in patients with biochemical persistence and 0.49 ng/ml in patients with biochemical recurrence.

### Factors predicting ^68^Ga-PSMA PET/CT positive findings

**Fig. 1:**
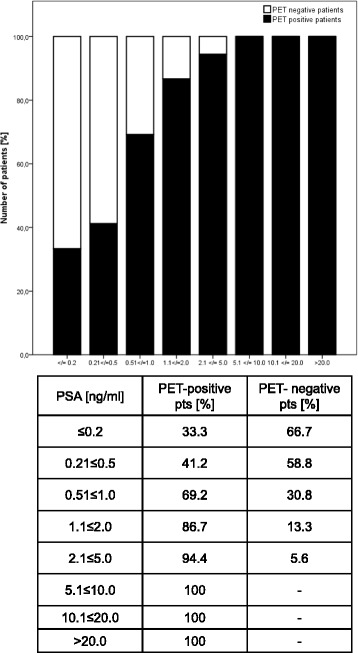
Probability of a pathological ^68^Ga-PSMA PET/CT depending on PSA levels in 129 patients. Corresponding table shows the rates of PET/CTs with/without pathological radiotracer uptake in % according to PSA level

**Fig. 2:**
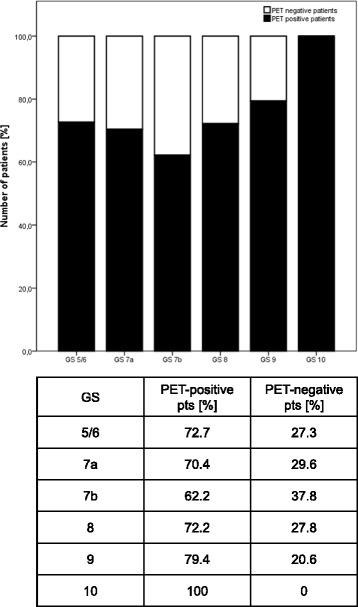
Probability of a pathological ^68^Ga-PSMA PET/CT depending on GS in 129 patients. Corresponding table shows the rates of PET/CTs with/without pathological radiotracer uptake in % according to GS class

Patients with pathological radiotracer uptake (*n* = 92; Table 2) had a median PSA of 1.90 ng/ml (range 0.14–150.0), a PSA doubling time of mainly ≤10 months (77.8%), mainly a T3 N0 prostate cancer, a predominant GS 9 (29.3%) and ongoing ADT at time of PET in 12 patients. They were injected with a mean activity of 191.96 MBq ^68^Ga-PSMA-HBED-CC (range 87–293) and were mainly high-risk patients (81.5%). Patients without pathological findings (*n* = 37; Table 2) had a median PSA of 0.30 ng/ml (range 0.13–3.24), a PSA doubling time of mainly ≤10 months (67.6%), mainly a T3 N0 prostate cancer and a predominant GS 7b. Mean activity of ^68^Ga-PSMA-HBED-CC radiotracer was 186.78 MBq (range 94–293) in PET-negative patients with patients being mostly high-risk patients (75.7%) and two patients having ongoing ADT at the time of PET scan. In the univariate analysis (Table 3) no significant difference was found between PET-positive and PET-negative patients regarding the use of ADT at time of PET (*p* = 0.222), the injected amount of ^68^Ga-PSMA-HBED-CC (*p* = 0.590), the PSA doubling time ≤/> 10 months (*p* = 0.517), GS (*p* = 0.285) and T- (*p* = 0.982) and N-category (*p* = 0.987). Concerning PSA before PET, a significant difference was found (*p* < 0.001). In the multivariate analysis, the significant association between a PET-positive result and PSA level a time of PET persisted (*p* = 0.002). Figures 1 and 2 demonstrate the probability of pathological ^68^Ga-PSMA PET/CT depending on PSA level at time of PET/CT and GS. There is an almost linear increase of PET-positive result with rising PSA level: Patients with PSA ≤ 0.2 ng/ml had a detection rate of 33.3%, patients with PSA 0.21 ≤ 0.5 ng/ml a rate of 41.2% and patients with PSA 0.51 ≤ 1.0 ng/ml a rate of 69.2%.

**Table 2:** Patient characteristics: PET positive vs. PET negative

Characteristic	PET positive	PET negative
Number	92	37
Age (years) at PSMA PET (median, range)	72 (49–86)	72 (47–80)
Gleason Score [pts]		
6	8 (8.7%)	3 (8.1%)
7a	19 (20.7%)	8 (21.6%)
7b	23 (25.0%)	14 (37.8%)
8	13 (14.1%)	5 (13.5%)
9	27 (29.3%)	7 (18.9%)
10	2 (2.2%)	–
Risk group (D’Amico) [pts]		
low	3 (3.3%)	2 (5.4%)
intermediate	14 (15.2%)	7 (18.9%)
high	75 (81.5%)	28 (75.7%)
PSA PRE-PSMA PET [ng/ml] (median/mean, range)	1.90/8.27 (0.14–150.0)	0.30/0.50 (0.13–3.24)
PSA doubling time		
≤ 10 months	56 (77.8%)	25 (67.6%)
> 10 months	16 (22.2%)	12 (32.4%)
ADT at time of PET [pts]	12 (13.0%)	2 (5.4%)
Activity of 68Ga-PSMA-HBED-CC [MBq] (mean, range)	191.96 (87–293)	186.78 (94–293)

(*Pts* patients, *PSA* prostate specific antigen, *ADT* androgen deprivation therapy, *MBq* Mega-Becquerel)

**Table 3:** Univariate and multivariate analysis of factors predicting 68Ga-PSMA PET/CT positive findings

Association between PET positive results and	Present (yes/no)	*p*-Value^a^	*p*-Value^b^
ADT	No	0.222	0.321
Activity of 68Ga-PSMA-HBED-CC [MBq]	No	0.590	0.843
PSA PRE-PSMA PET	Yes	<0.001*	0.002*
PSA doubling time ≤/> 10 months	No	0.517	0.942
Gleason Score	No	0.285	0.482

(*PSA* prostate specific antigen, *ADT* androgen deprivation therapy, *MBq* Mega-Becquerel)

^a^univariate and ^b^multivariate binary logistic regression analysis

^*^p < 0.05 statistically significant

### Tumour location and PET versus CT-positive findings

**Fig. 3:**
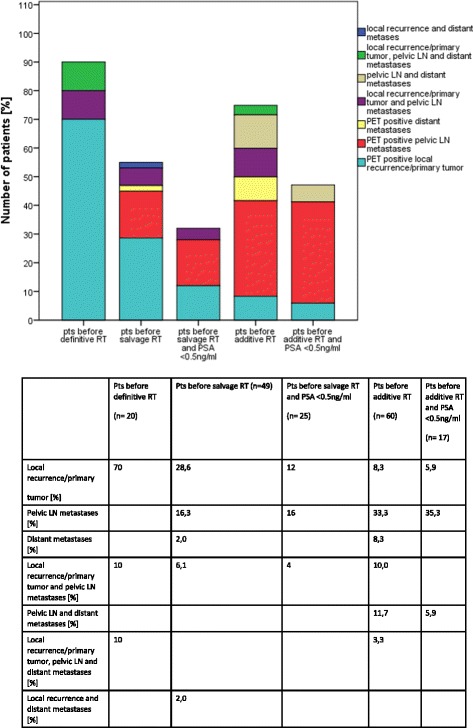
Lesion based analysis (PET-positive local recurrence/primary tumour and PET-positive lymph node and distant metastases) according to sub-grouping of patients (patients before definitive RT at initial diagnosis, patients before salvage RT with PSA recurrence, patients before Salvage RT with PSA < 0.5 ng/ml, patients before additive RT with PSA persistence, patients before additive RT with PSA persistence with PSA < 0.5 ng/ml)

**Fig. 4:**
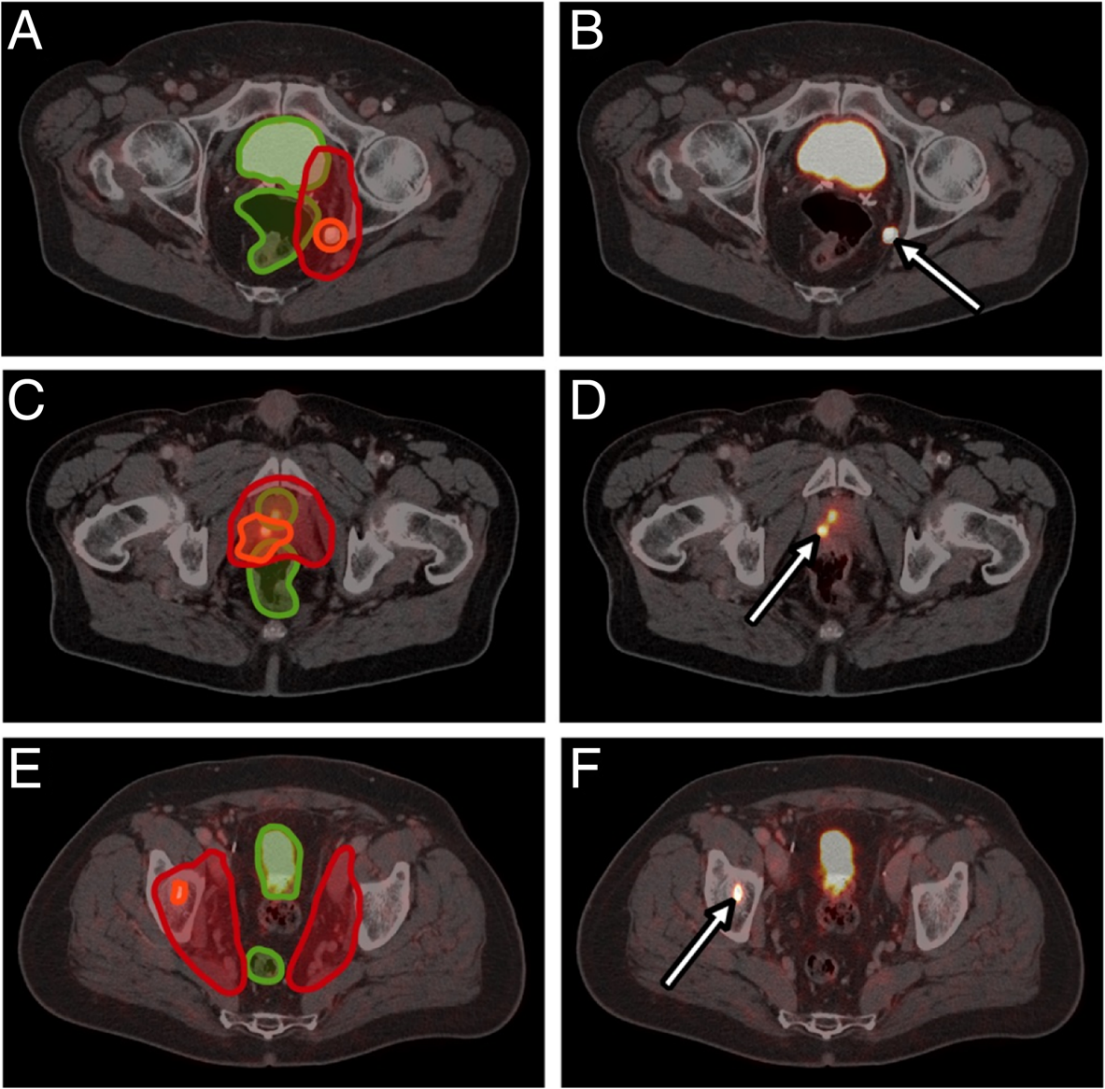
Radiotherapy treatment planning in three patients based on PSMA PET/CT information. ^68^Ga-PSMA PET/CT (**B**, **D**, **F**) and target volumes with simultaneously integrated boost volumes (**A**, **C**, **E**) are shown. Figs. **A** and **B** show a patient with PSA recurrence and evidence of lymph node metastasis in ^68^Ga-PSMA PET/CT who was treated with a simultaneously integrated boost to the lymph node. Figs. **C** and **D** show a patient with PSA recurrence due to local relapse in the region of the former prostate gland. This local relapse was treated with a simultaneously integrated boost volume. Figs. **F** and **E** show a patient with singular osseous metastasis that was treated with a simultaneously integrated boost

Prior to definitive radiotherapy, ^68^Ga-PSMA PET/CT showed suspicious lesions within the prostate gland in 90% of patients and suspicious pelvic lymph nodes and/or distant metastases in 20%/10% of the patients, respectively. ^68^Ga-PSMA PET/CT had a high detection rate of prostate cancer recurrence outside the prostatic fossa in patients being considered for salvage radiotherapy: 22.4% of patients had PET-positive pelvic lymph nodes and 4.1% distant metastases. Both patients with distant metastases had also local recurrence exclusively without evidence of pelvic lymph node metastases. In patients considered for salvage radiotherapy with a PSA < 0.5 ng/ml (25 patients), ^68^Ga-PSMA PET/CT detected in 16.0% local recurrences within the prostatic fossa and in 20.0% PET-positive pelvic lymph nodes. In patients with postoperatively biochemical persistence, PET/CT revealed a high number of lymph node metastases (58.3%), distant metastases (23.3%) and macroscopic residual tumour (21.7%). Seventeen of 60 patients with persistent PSA after radical prostatectomy had a pre-PSMA PET/CT PSA <0.5 ng/ml. Of these patients, there was one patient (5.9%) with macroscopic residual tumour, 7 patients with pelvic lymph node metastases (41.2%) and 1 patient with distant metastases (5.9%). The single patient with bone metastases had pelvic lymph node involvement as well. Figure 3 shows a lesion based analysis of PET-positive local recurrence/primary tumour, PET-positive lymph node and distant metastases according to sub-grouping of patients and contains additional information on simultaneous involvement of local recurrent tumour/primary tumour, lymph node metastases and/or distant metastases. ^68^Ga-PSMA PET/CT as medical imaging modality had a significantly higher diagnostic value in the detection of suspicious lesions within the prostate/prostatic fossa (38% vs. 10.1%; *p* < 0.05), of suspicious pelvic lymph nodes (38.8% vs. 15.5%; p < 0.05) and distant metastases (14.0% vs. 5.4%; p < 0.05) compared to the contrast enhanced CT scan acquired during PET/CT imaging. This resulted in a modification of radiotherapy treatment in 56.6% of patients by e.g. enlarged target volumes or simultaneously integrated boost volumes. Figure 4 demonstrates the integration of ^68^Ga-PSMA PET/CT images into radiation therapy planning in three patients.

## Discussion

This heterogeneous group of patients with primary, recurrent or persistent prostate cancer represents a typical cohort referred to radiation oncology departments. We are thus confident that our findings are of relevance in clinical practice.

In general, out of 129 patients with high-risk primary, persistent or recurrent prostate cancer, PSMA-PET revealed a least one relevant lesion in 71.3% of the cohort. This rate compares nicely to the number of PET-positive patients (82.8%) in the study of Afshar-Oromieh et al. [16]: a similarly heterogeneous group of patients with progressive disease after initial treatment (e.g. radiotherapy and/or surgery; *n* = 292), before initiation of local therapy (*n* = 27) or before therapy with radiolabelled PSMA ligands (*n* = 38) was analysed.

The clear correlation of PSA level and probability of detection was seen in our analysis (Fig. 1) as well as in the data provided by Ceci et al. [25] (*n* = 70), Afshar-Oromieh et al. [16] (n = 292) and Eiber et al. [17]. Accordingly, patients with biochemical recurrence having the lowest PSA levels prior to PSMA PET/CT had the lowest detection rate (55.1%) compared to patients with persistent (75%) or primary prostate cancer (100%).

At present, PSA levels ≤0.2 ng/ml are frequently judged in postoperative patients to be non-critical, nevertheless one-third of our patients (33.3%) had PET-positive findings even below a PSA of 0.2 ng/ml. This rate drastically increased up to 69.2% in patients with a PSA level between 0.51–1.0 ng/ml. Thus, PSMA PET/CT detects early local recurrence or metastatic disease and possibly allows for a more effective and early treatment.

Apart from PSA-level before PET/CT, multivariate analysis detected no significant associations between a positive PSMA PET/CT and any of the other factors including ADT, amount of injected tracer, PSA doubling time, and Gleason score. This finding is well in accordance with the observations provided by Afshar-Oromieh et al. [16] regarding PSA doubling time and Gleason score. In contrast, Afshar-Oromieh et al. reported a positive association between detection rate and ongoing ADT. In animal models and in vitro cell culture experiments, ADT increases PSMA expression which might contribute to improved detection rate by ^68^Ga-PSMA PET/CT [26–30]. However, at present the complex association between an ADT driven increase of PSMA uptake in the individual tumour lesion, the ADT driven reduction of global tumour load and the final PET signal is not completely understood. In our opinion, the use of ADT in a cohort with mostly high-risk prostate cancer patients as evaluated by Afshar-Oromieh et al. is also a surrogate for high tumour burden and high PSA levels. We therefore counsel our patients to start with ADT after PSMA PET/CT if planned. Unlike the aforementioned trials, Ceci et al. also observed a significant association between PSA doubling time and PSMA PET/CT positivity [25]. This might be explained by the fact that Ceci et al. coded PSA doubling time as continuous variables whereas in our trial and the trial of Afshar-Oromieh et al. PSA doubling time was analysed in distinct categories.

Compared to CT, sensitivity of PSMA PET/CT was significantly higher for local findings (38.0% vs. 10.1%), pelvic lymph nodes (38.8% vs. 15.5%) and distant failure (14.0% vs. 5.4%). This observation perfectly mirrors the findings reported by Giesel el al. showing that PSMA PET/CT is significantly more sensitive than CT-based 3D–volumetric lymph node evaluation [18].

Furthermore, we analysed the question how far an increased detection rate leads to a change in therapeutic management. In our series, PSMA PET/CT-positive findings had a substantial influence on the therapeutic concept in 56.6% of the patients with implication on either adaptation of treatment volumes, dose concepts or the commencement of hormone ablation therapy. This compares nicely to similar analyses [19, 31–33] all reporting major modifications of therapeutic management in 33.3% - 53.7% of the patients.

At present, PSMA PET/CT is widely regarded as the best modality for lymph node staging in a primary as well as in a postoperative setting [14, 34]. Data provided by van Leeuwen et al. [19] suggest that the level of lymph node positivity has been strongly underestimated in the pre-PSMA PET/CT era. In their analysis, lymphatic recurrences outside the prostatic fossa occurred in 20% of patients scheduled for salvage radiotherapy with additional 10% of distant metastases detected in PSMA PET/CT. Bearing in mind that currently only target volumes covering the prostatic fossa are routinely employed [35], a substantial rate of lymph node metastases is not treated. This is supported by our own data showing that even below a PSA level of 0.5 ng/ml, PSMA PET/CT still detected 20.0% of pelvic lymph node metastases in patients with salvage radiotherapy indication.

The documented need to treat PSA relapses as early as possible (even below PSA levels of 0.2 ng/ml) and the fact that the diagnostic sensitivity is optimal above a PSA level of 0.5 ng/ml currently creates an inherent dilemma. Since all data available consistently document that PSA control is significantly better when radiotherapy is commenced as early as possible [36, 37], it is not justified to wait until PSA is in an optimal diagnostic range. Our strategy to cope with this dilemma is therefore to employ PSMA PET/CT in patients even around 0.2 ng/ml especially whenever high-risk features are present. Like the analysis by Henkenberens et al. on 29 patients with biochemical recurrence receiving an individualized radiotherapy treatment plan based on PSMA PET/CT findings [38], target volumes are modified accordingly in our institution after critical assessment. Nevertheless, keeping the sensitivity and specificity rates from surgical series in mind, PSMA PET/CT might still underestimate the true extent of disease and should therefore from our point of view not result in an omission of nomogram [39] triggered radiotherapy treatment volumes.

At present, legal constraints prohibit the use of PSMA in truly prospective studies in Germany. Despite the clear limitations of retrospective approaches, the available data from several analyses strongly underline the high value of PSMA-PET/CT for staging as well as for treatment stratification in patients with primary high-risk, persistent or recurrent carcinoma of the prostate.

## Conclusions

In this retrospective study, we showed that the detection of prostate cancer is strongly associated with PSA level at time of Ga-PSMA PET/CT. Better than any other imaging modality, PSMA PET/CT differentiates local, regional and distant metastatic disease with considerable implications for disease management. In post-prostatectomy patients with rising PSA ≤ 0.5 ng/ml with indication for salvage radiotherapy, PSMA PET/CT is narrowing the diagnostic gap as until now the gradual PSA increase often occurred long before recurrent disease could be localized clinically or by imaging.

## Abbreviations

ADT: androgen deprivation therapy
GS: Gleason score
MBq: megabecquerel
PCa: prostate cancer
PET: positron emission tomography
PET/CT: positron emission tomography/computed tomography
PSMA: prostate-specific membrane antigen
Pts: patients
RT: radiotherapy
